# Cases of Foreign Bodies in the Larynx and Trachea

**Published:** 1829-04-01

**Authors:** 


### XXIX.

### CASES OF FOREIGN BODIES IN THE LARYNX AND TRACHEA.

Dr. J. Reiche has published two interesting cases of this kind, in a German cotemporary,* for the purpose of shewing the great similarity between the symptoms thus produced and those of croup, as well as pointing out some diagnostic marks.

*Case* 1. On the 7th Dec. 1824, Dr. R. was summoned to see a little girl, ætatis 2½, whom he found playing in her bed, and apparently well. The breathing, however, was a little hurried, and, every quarter of an hour, the child took an inspiration so slow as to occupy fifteen or twenty seconds, and accomplished with the most extraordinary effort and distress. The chiid appeared dying, and yet the succeeding expiration brought a perfect calm, only interrupted by a slight cough, without expectoration, or any thing other-

* Rust's Magazin für die gesummte Heilkunde; 27 vol. per Cahier, 1828.

wise particular. Nothing could be seen in the throat or fauces, but the whole anterior part of the neck and chest were emphysematous, so much so, that the larynx and trachea could with difficulty be felt. On applying the ear to the chest, the respiratory murmur was not heard at all at the upper part of the right side, and below it was feeble. Between the cartilages of the second, third, and fourth ribs, the "râle sifflant" was heard. The voice was shrill, the pulse soft and slow. From these symptoms, Dr. Reiche assured the parents, that, in all probability, a foreign body was lodged in the child's trachea, and, on strictly questioning the servant-maid, it was found that, on the preceding evening, she had put into its mouth a quarter of a nut whilst crying, immediately after which, it was seized with so violent a fit of coughing as to threaten suffocation. After a time the paroxysm went off, and the parents of the child remarked nothing particular about it, save that it was restless, and wheezed a little in its breathing. Towards nine o'clock that evening, the child, however, became so ill that a medical man was sent for, who considered the case as one of croup, and ordered leeches to the neck, emetics, &c. On the next morning the emphysema made its appearance, and, at noon, when our author was summoned in consultation, the symptoms were those which have already been described.

Both medical gentlemen were now convinced that, without tracheotomy, the patient must die, and stated as much to the parents. These latter however, obstinately refused to allow of its performance, influenced by a case related by the grandmamma, where a young peasant had coughed up a pea which was lodged in the windpipe. The grandmamma prevailed over the doctors, and all that was left for the latter to do was, to palliate the bronchial irritation and inflammation by leeches to the right side, demulcents, and a powder of calomel and extract of hyosciamus, together with an emetic, to second the natural efforts at expulsion of the foreign body. During the night, no aggravation of the symptoms took place, and no relief had ensued on the 9th. The cough was now accompanied with expectoration, which the child invariably swallowed. In the evening the cough was so severe as to excite convulsions, the paroxysms of difficult inspiration returned every five or six minutes, the wheezing was very loud, the emphysema had extended. Calomel, ext. hyosciam. digitalis, radix ipecacuanhæ, and the sulphuret of antimony, julep of the syrup of senega and bitter almond-water, and a blister, were the means employed. On the 10th, after a convulsive paroxysm of coughing, a calm ensued, and the child fell asleep. The râle sifflant now disappeared—in the afternoon, the portion of undigested nut was discovered in the stools—the little patient improved daily—the emphysema, in a fortnight or three weeks, had vanished, and, in short, the patient speedily recovered.

Dr. Reiche supposes, and no one can reasonably doubt the fact, that during the violent coughing, the child had ejected the portion of nut from the respiratory tube, and swallowed it with the rest of the expectorated matters. Although the grandmother proved to be right, yet the medical consultants were fully justified in proposing the operation, if not in hazarding the decided opinion they did on the probable consequence of its not being resorted to. The emphysema that took place is always to be looked on as a formidable symptom, though some degree of difficulty must arise in determining its immediate cause. In the celebrated case recorded by M. Louis, in the Mémoires of the French Academy of Surgery, it depended, not on laceration of the mucous membrane of the trachea, but in rupture of the air-cells of the lungs themselves, and escape of the air from thence to the anterior mediastinum and neck. Whether this was the case in the present instance, we leave to our readers to settle as they please.

*Case* 2. On this we shall touch very briefly. The patient was a girl, six years of age, who swallowed a plum-stone, and was instantly seized with a fit of suffocation, which returned from time to time, accompanied with violent cough and expectoration, mixed with streaks of blood. She was seen, next day, by Dr. Reiche, who found nothing but the râle sifflant during respiration, a frequent cough, and a want of the respiratory murmur betwixt the second and third costal cartilages on the right side. There were neither those occasional difficult inspirations, nor the emphysema noticed in the former case. The trachea was opened from its second to its sixth ring, but the foreign body could not be found. The introduction of an oiled sound into the wound occasioned such pain that it was given up, and the patient conveyed to bed. In the course of half an hour, our author made some fresh attempts to discover and extract the stone, and at length his exertions were crowned with success, the foreign body being removed by the common forceps. The wound was a considerable time in healing, but the little patient ultimately did well.

Dr. Reiche remarks that, in both these cases, and in most of those that he has read in authors, the whistling (*sifflement*) of the breathing was remarkably distinct. When coupled with the other symptoms, and the circumstances under which they made their appearance, he thinks it would be sufficient to point out the nature of the accident, even although no mention should be made of a foreign body having been swallowed. The symptoms are most easily, indeed most frequently, confounded with croup; but, setting aside the absence of the "bruit *sifflant*" in that disease, Dr. R. believes that the frequent cough, together with the alteration of sound, both in it and in the voice, would sufficiently distinguish the symptoms of croup, from those of the lodgement of foreign bodies in the larynx or trachea.

